# Evaluation of chemical and microbial quality of food in northern Iran

**DOI:** 10.1186/s41043-021-00266-7

**Published:** 2021-09-20

**Authors:** Zahra Aghalari, Seyed Reza Hosseini, Somayeh Jafarian, Mohsen Rezazadeh, Mohsen Mirzaei, Ebrahim Esmaeili, Peyman Hasanzadeh

**Affiliations:** 1 Social Determinants of Health Research Center, Health Research Institute, Babol University of Medical Sciences, Babol, Iran; 2 Health Services Management, Environmental Health Engineering, Health Department, Social Determinants of Health Research Center, Health Research Institute, Babol University of Medical Sciences, Babol, Iran; 3 Deputy of Food and Drug, Babol University of Medical Sciences, Babol, Iran; 4 Director of the Center for Environmental and Occupational Health, Deputy of Health, Babol University of Medical Sciences, Babol, Iran

**Keywords:** Food hygiene, Food quality, Microbial and chemical contamination

## Abstract

**Background:**

Iran is one of the developing countries and foodborne diseases commonly impose problems for public health, the health care system and the economy. Therefore, this study aimed to evaluate the chemical and microbial quality of food in northern Iran.

**Methods:**

This cross-sectional study was conducted in 2019. This study was performed on food samples obtained in a straightforward way while visiting food preparation and distribution centers in Babol. Tests related to different food types were performed by laboratory experts. Data collection with a checklist: date and place of sampling, number of samples, type of food, type of test, compliance of results with standards. Data were analyzed by SPSS_22_ and descriptive statistics, Chi-square and *t*-test.

**Results:**

1043 food samples were tested from 5 groups of dairy products, protein, cereals, vegetables and other food groups. The highest number of samples in the cereal group was 767 samples (73.53%). In the cereal group, most samples were breads. The pH of 11.67% of breads and the salt in 21.49% of breads did not match the standard. The blankit (sodium hydrosulfite) on bread dough were negative. Moisture, gluten, ash and pH match with the standards in all flour samples. The results of microbial tests on sweets and ice cream showed that *Escherichia coli* and *Staphylococcus aureus* and *Salmonella* were negative. *Enterobacter aerogenes* was positive in 8.20% of sweets, mold and yeast were positive in 19.58%. The results of microbial tests on buttermilk and yogurt, grilled meat and chicken sandwiches, vegetables and salads showed that bacteria *Escherichia coli* and *Staphylococcus aureus* and all microorganisms were negative. Mold tests were positive in 11.12% of juice samples.

**Conclusions:**

The results showed that the foods and drinks supplied in food and drink preparation and distribution centers in Babol in 2019 were of good chemical and microbial quality. In some food groups the results of microbial and chemical testings were negative, i.e. without contamination. Less than 20% of products in the group of cereals and protein products did not match with the standards, which is a satisfactory result compared to other studies conducted in different cities of Iran. These good results for food quality can be explained bythe constructive performance of food health experts that made good controling, monitoring, and food health and hygiene education.

## Introduction

Food safety and hygiene means preventing food contamination with a variety of chemical toxicants such as heavy metals, pesticides, bacteria, viruses, parasites and fungi [1, 2]. The World Health Organization identifies diseases caused by food contamination as a most important recent public health problem worldwide [3]. In this regard, the slogan of 2018 environmental health was named (Global Food Safety and Sustainability) by the Information Federation of Environmental Health [4]. Factors involved in the foodborne diseases epidemic include inadequate food storage and environmental factors such as temperature and humidity, contamination of tools, preparation of food from unhealthy sources, poor personal hygiene and insufficient cooking time [5]. Common symptoms of food-borne diseases include diarrhea, fever, headache, vomiting, abdominal cramps, extreme tiredness, and sometimes blood in the stool [6, 7].

In developing countries such as Iran, however, there are no statistics on the incidence of food poisoning and infections. It can be expected that due to improper production, storage, distribution, and consumption of food, the prevalence of food poisoning and foodborne diseases is much higher than in developed countries. Iran is one of the developing countries. This study aims to evaluate the chemical and microbial quality of food in Northern Iran.

## Methods

This article is cross-sectional study. Food sampling was performed by environmental health experts while visiting food preparation and distribution centers, including restaurants and grocery stores in urban and rural areas of Babol in 2019. Babol City located in Northern Iran and 18 km from the Caspian Sea with a population of 531,930, is the most populous city in the Province of Mazandaran. The height of the city is 2 m sea level [8].

After taking the samples, the environmental health experts transferred them to the laboratory of the Food and Drug Administration in Babol. Tests related to each type of food were performed by laboratory experts according to the type of chemical and microbial tests and applying standard methods [9].

The research tool was a checklist to record food information. Food information such as: date and place of sampling, number of samples, type of food (dairy products, protein, cereals, vegetables and other food groups), food product subgroups (cereals: flour, bread, dry and wet sweets), (dairy products: milk, yogurt, ice cream and buttermilk), (protein products: kebabs, meat sandwiches, chicken), (vegetables: salads and vegetables consumed in restaurants), type of test (microbial and chemical), types of chemical tests including (pH, salt, color, acidity and polarity), types of microbial tests were including (mold, yeast, *Salmonella, Escherichia coli and Staphylococcus*, all microorganisms), match of results with National standards [9]. A number of food samples (*n* = 50) were removed from the checklist due to inconsistency with the research objectives and incomplete information.

Data were analyzed by SPSS_22_ and descriptive statistics, Chi-square, and *t*-test.

## Results

In this study, 1043 food samples were tested in 5 groups of dairy products, protein, cereals, vegetables and other food groups. The highest number of samples in the cereals group was 767 samples (73.53%). In the cereals group, most samples were breads.

### Results of chemical and microbial tests in cereal products

540 breads were collected from urban (39.44%) and rural (60.56%) bakeries in Babol. The mean pH of breads was 5.96 ± 0.46, the lowest and highest pH in breads were 5 and 9.60, respectively. The mean pH of breads in urban was 6.29 ± 0.35 and in rural was 5.61 ± 0.52. *T*-test showed that the mean pH was not significantly related to bread sampling areas (*P* = 0.451, *T* =  − 1.285).

The mean salt in breads was 0.91 ± 0.51, the lowest and highest salts in breads were 0.11 and 4.09, respectively. The mean salts of breads in urban areas was 0.90 ± 0.52 and in rural areas was 0.92 ± 0.50. *T*-test showed that the mean salt was significantly associated with breads sampling areas (*P* = 0.048, *T* =  − 0.419).

Breads in percentages of 88.33% and 78.51% were of acceptable quality within the standards in terms of pH and salt content, respectively. pH in 11.67% of breads and salt in the 21.49% of breads did not match the standard. The type of bread had a significant relationship with the positive results of pH, so that the pH of Barbari bread was higher. The amount of salt with sampling seasons and the amount of salt had a significant relationship with the sampling area, so that the amount of salt was higher in summer and in rural areas (Table 1).

**Table 1:** Results of chemical tests of bread in Babol-2019

Variables	Levels	pH test results		*χ*^2^	*P* value	Salt test results		*χ*^2^	*P* value
		Positive	Negative			Positive	Negative		
Type of bread	Barbari	32 (8)	367 (92)	36.643	0.001	92 (23.1)	307 (76.9)	4.879	0.181
	Taftoon	26 (31)	58 (69)			18 (21.4)	66 (78.6)		
	Lavash	2 (15.4)	11 (84.6)			2 (15.4)	11 (84.6)		
	Sangak	3 (6.8)	41 (93.2)			4 (9.1)	40 (90.9)		
Sampling seasons	Spring	23 (30.3)	53 (69.7)	34.192	0.001	21 (27.6)	55 (72.4)	39.525	0.001
	Summer	23 (9.5)	219 (90.5)			76 (31.4)	166 (68.6)		
	Autumn	5 (3.9)	123 (96.1)			7 (5.5)	121 (94.5)		
	Winter	12 (12.8)	82 (87.2)			12 (12.8)	82 (87.2)		
Sampling area	Urban	28 (13.1)	185 (86.9)	0.746	0.388	30 (14.1)	183 (85.9)	11.410	0.001
	Rural	35 (10.7)	292 (89.3)			86 (26.3)	241 (73.7)		

The blankit (sodium hydrosulfite) test of bread dough were negative. The mean ash test on 22 flour samples was 0.72 ± 0.08 g/100 mL. The lowest and highest ash levels in flours were 0.5 g/100 mL and 0.88 g/100 mL, respectively. The mean of gluten in flour samples was 28.209 ± 1.319. The lowest and highest gluten in flours were 26 and 30, respectively. The mean pH in flour samples was 6.24 ± 0.10. The lowest and highest pH in flours were 6 and 6.4, respectively. The mean moisture of flour samples was 11.84 ± 0.96 g/100 mL. The lowest and highest moisture in flours were 10.20 g/100 mL and 12.77 g/100 mL, respectively. The moisture in the flours was significantly related to the sampling area with moisture of flours being higher in urban areas (Table 2).

**Table 2:** Results of chemical and microbial tests on bread dough and flour in Babol-2019

Variables	blankit test Mean ± SD		*F*	*P* value
	Urban	Rural		
*Type of test: results of chemical on blankit test of bread dough*				
Ash g/100 mL	0.731 ± 0.729	0.715 ± 0.963	0.321	0.577
Gluten	28.542 ± 1.509	28.053 ± 1.245	0.116	0.737
pH	6.224 ± 0.108	6.259 ± 0.102	0.22	0.883
Moisture g/100 mL	12.371 ± 0.298	11.602 ± 1.074	32.505	0.001

Variables	Levels	Mold test results		*χ*^2^	*P* value
		Positive	Negative		
*Type of test: results of microbial on Flour samples*					
Sampling seasons	Spring	5 (35.7)	9 (64.3)	2.335	0.506
	Summer	2 (28.6)	5 (71.4)		
	Autumn	1 (50)	1 (50)		
	Winter	0	4 (100)		
Sampling area	Urban	2 (18.2)	9 (81.8)	1.167	0.280
	Rural	6 (37.5)	10 (62.5)		

Flour samples (*n* = 27) were tested for molds and other microorganisms. The test results showed that none of the flour samples tested positive for all microorganisms. There was no mold in 70.37% of flours (Table 2).

Color test was performed on 59 samples of sweets. There was no color in 44 samples (74.57%) and only 15 samples (25.43%) had color. Color in the sweets had a significant relationship with sampling seasons (Table 3).

**Table 3:** Results of chemical and microbial tests on sweets in Babol-2019

Variables	Levels	Color test		*χ*^2^	*P* value
		Positive	Negative		
*Type of test: results of color in sweets*					
Sampling seasons	Spring	9 (36)	16 (64)	8.315	0.040
	Summer	1 (6.7)	14 (93.3)		
	Autumn	0	7 (100)		
	Winter	5 (41.7)	7 (58.3)		
Sampling area	Urban	4 (17.4)	19 (82.6)	1.283	0.257
	Rural	11 (30.6)	25 (69.4)		

Variables	Levels	Mold		*χ*^2^	*P* value	Yeast		*χ*^2^	*P* value
		Positive	Negative			Positive	Negative		
*Type of test: results of microbial tests on sweets*									
Type of sweets	Dry	10 (11.8)	75 (88.2)	26.696	0.001	9 (10.6)	76 (89.4)	35.329	0.001
	wet	9 (75)	3 (25)			10 (83.3)	2 (16.7)		
Sampling seasons	Spring	2 (13.3)	13 (86.7)	6.967	0.073	2 (13.3)	13 (86.7)	6.967	0.073
	Summer	9 (29)	22 (71)			9 (29)	22 (71)		
	Autumn	8 (24.2)	25 (75.8)			8 (24.2)	25 (75.8)		
	Winter	0	18 (100)			0	18 (100)		
Sampling areas	Urban	4 (9.1)	40 (90.9)	5.633	0.018	3 (6.8)	41 (93.2)	8.337	0.004
	Rural	15 (28.3)	38 (71.7)			16 (30.2)	37 (69.8)		

The results of microbial tests on 97 sweets showed that *Escherichia coli* and *Staphylococcus aureus* and *Salmonella* were negative. The results of *Enterobacter* tests were positive in 8 samples (8.24%), mold and yeast were positive in 19 samples (19.58%) (Table 3).

### Results of chemical and microbial tests in dairy products

Out of 31 samples of ice cream and juices, 90.32% had not color and only 9.68% of the samples had color.

The results of microbial tests on 32 ice creams showed that *Escherichia coli* and *Staphylococcus aureus* were negative. *Salmonella* was negative in 31 ice cream (96.87%) whereas it was detected in only 1 sample (3.13%). Total microorganisms were negative in 23 samples (71.87%) and positive in 9 samples (28.13%).

Microbial tests on 18 juices showed that *coliforms*, total microorganisms, *Salmonella*, *Staphylococcus aureus*, *Escherichia coli* and yeasts were negative in the juices. Mold was negative in 16 juices (88.88%) and mold was detected in only 2 samples (11.12%).

Microbial tests on 30 buttermilk and yogurt types showed that *Escherichia coli* and *Staphylococcus aureus* and total microorganisms were negative.

### Results of chemical and microbial tests in protein products

Out of 31 samples of meat stews and grilled chicken, 22 samples (71%) had no color. 23 samples of meat sandwiches and grilled chicken were negative for *coliforms*, total microorganisms, *Salmonella*, *Staphylococcus aureus* and *Escherichia coli*. The mold test was negative in 67.74% of meat samples and positive in 32.26%. Chi-square showed that different seasons had a significant relationship with mold positivity in meat samples (*P* = 0.034, *χ*^2^ = 8.695), the most positive results of mold in meat samples were in summer.

### Results of microbial tests on vegetable products

Microbial tests on 9 samples of vegetables and salads from restaurants showed that all samples were negative for mold, *coliforms*, total microorganisms, *Salmonella*, *Enterobacter, Staphylococcus aureus* and *Escherichia coli*.

### Results of chemical and microbial tests on other food products

Acidity and total polar material (TPM) tests were performed on 125 samples of frying oil used in confectioneries and restaurants. Tests on 113 oil in confectionery samples showed that the mean acidity was 0.14 ± 0.15 g/100 mL, the minimum and maximum acidities were 0.02 g/100 mL and 0.93 g/100 mL, respectively. The mean TPM was 11.63 ± 3.10 mg, the minimum and maximum TPM were 8 and 23.50, respectively.

Tests on 12 oils used in restaurants showed that the mean acidity in oils was 0.21 ± 0.212 g/100 mL, the minimum and maximum acidity were 0.02 g/100 mL and 0.64 g/100 mL, respectively. The mean TPM was 12.54 ± 4.01 mg, the minimum and maximum TPM were 8 mg and 23.50 mg, respectively. The results of acidity and TPM tests on all oil samples taken from confectioneries and restaurants were negative.

## Discussion

In this study, Bread as the dominant food has a major share in the consumption pattern of households in Iran. The per capita consumption of wheat and bread in Iran is about 24 and 300 kg, respectively [10, 11], so most of the food samples were breads. In this study, the pH was ≤ 6 and for salt was less than one in breads. According to national standards, pH (≤ 6) and salt (≤ 1.8%) are acceptable [9]. 78.51% of breads had no problem with salt and were matching the standards. In a study by Hagh Nazari et al. (2016) 5% of breads had unsuitable pH and there was baking soda in breads [12]. In a study by Malakotian et al. (2003) showed that the pH and salt of breads were 5.4 g and 3.27 g, respectively [13]. In this study, the pH level in 11.67% of breads were beyong permissable values, compared to the breads sampled in Rafsanjan, 22.33% (13), Yazd breads 12% [14], had a pH beyond the standard, the results of the present study can provide good evidence that in the city of Babol, health experts have done successful work in controling, monitoring and providing health education.

In the present study, the mean moisture of the flours was 11.84 ± 0.96 g/100 mL and the mean ash was 0.72 ± 0.08 g/100 mL. A study by Khoshakhlagh et al. (2015) showed that the mean moisture of flour was 14 ± 0.004 g/100 mL and the mean ash was 1.32 ± 0.06 g/100 mL [15]. A study by Nasehi et al. (2014) showed that the amount of ash in some samples of flour was more than the standard [16]. But in our study, the amount of ash in all flour samples was within the standards, indicating the production of quality bread in Babol.

In the present study, 25.43% of the sweets had color. In this study, 9.7% of ice cream and juices had color. A study by Rahimi et al. (2016) showed that 50.4% of food samples had color [17]. In a study by Asadi et al. (2019) showed that artificial colors were used in 42.9% of the foods offered in restaurants in Fasa [18]. Comparing the results of this part of the study with other mentioned studies shows that the food in Babol was in good condition and only a small percentage of the samples had color. But because some artificial colors in small quantities cause allergies and lead to tongue inflammation and hyperactivity disorders in children [19, 20], it is suggested that more monitoring should be done in this sector and violators should be fined.

In this study, the results of microbial testing of sweets showed that *Escherichia coli* and *Staphylococcus aureus* and *Salmonella* were negative. *Enterobacteriaceae* were positive in 8.20%, mold and yeast in 19.58% of sweets. Mold and yeast were more prevalent in wet sweets and rural areas. A study by Nasehinia et al. (2017) showed that 33.8% of the sweets had microbial contamination, of which mold and yeast contamination provided 21.7% and 11.4%, respectively [21]. A study by Nikniaz et al. (2011) showed that 48.8% of the wet sweets samples were contaminated by *Escherichia coli*, 38.8% with *coliforms*, 31.2% with *Staphylococcus aureus*, and 70% with yeasts [22]. In a study by Masoumalinejad et al. (2017) it was reported that high contamination of wet sweets sampled in spring was related to bacillus, mold and yeast and in summer these were related to yeast. In dry sweets, contamination was attributed to molds in spring, yeast and Bacillus in summer [23]. In the United Kingdom, more than 30% of foodborne diseases were related to confectionery, with *Staphylococcus* being the most common contamination [24]. Because fungal spores are scattered in the air, they can contaminate sweets. Contamination of confectionery containers, contamination of distributors and fungal contamination of confectionery materials such as sugar and flour can lead to mold and yeast contamination in sweets, so to prevent contamination of pastries with mold and yeast, hygienic conditions must be observed.

## Conclusion

The results showed that the food supplied in food preparation and distribution centers in Babol is of sufficient chemical and microbial quality, that in some food groups the results of microbial and chemical tests were completely negative. Less than 20% of products in the group of cereals and protein products did not match with National standards, which is a satisfactory result compared to other studies conducted in different cities of Iran. The good results related to food quality are explained by the fact Babol health experts in Babol have done a good control, monitoring and health education.

One of the strengths of this report is evaluation of chemical and microbial quality in different food groups. One of the study weaknesses were easy sampling and small number of food samples in some groups. The results of bread tests can be generalized to other bread samples due to the large volume of samples but the results of other food groups cannot be generalized.

## Data Availability

The datasets used and analysed during the current study are available from the corresponding author upon reasonable request.

## Abbreviations

WHO: World Health Organization
CDC: Centers for Disease Control and Prevention
UK: United Kingdom
SPSS: Statistical package for social science
TPM: Total polar material
